# Reducing the confusion and controversies around pragmatic trials: using the Cardiovascular Health Awareness Program (CHAP) trial as an illustrative example

**DOI:** 10.1186/s13063-015-0919-3

**Published:** 2015-09-02

**Authors:** Lehana Thabane, Janusz Kaczorowski, Lisa Dolovich, Larry W. Chambers, Lawrence Mbuagbaw, on behalf of the CHAP investigators

**Affiliations:** Department of Clinical Epidemiology and Biostatistics, McMaster University, Hamilton, ON Canada; Biostatistics Unit, Father Sean O’Sullivan Research Centre, St Joseph’s Healthcare, Hamilton, ON Canada; Departments of Paediatrics and Anaesthesia, McMaster University, Hamilton, ON Canada; Centre for Evaluation of Medicine, St Joseph’s Healthcare, Hamilton, ON Canada; Department of Family Medicine, McMaster University, McMaster Innovation Park, Hamilton, ON Canada; Population Health Research Institute, Hamilton Health Sciences, Hamilton, ON Canada; Department of Family and Emergency Medicine, Université de Montréal, Montreal, QC Canada; University of Montreal Hospital Research Centre (CRCHUM), Montreal, QC Canada; Centre for the Development of Best Practices in Health, Yaoundé Central Hospital, Yaoundé, Cameroon

**Keywords:** Pragmatic trials, Criticism, Knowledge translation, Cluster randomized trials

## Abstract

**Abstract:**

Knowledge translation (KT) involves implementation of evidence-based strategies and guidelines into practice to improve the process of care and health outcomes for patients. Findings from pragmatic trials may be used in KT to provide patients, healthcare providers and policymakers with information to optimize healthcare decisions based on how a given strategy or intervention performs under the real world conditions. However, pragmatic trials have been criticized for having the following problems: i) high rates of loss to follow-up; ii) nonadherence to study intervention; iii) unblinded treatment and patient self-assessment, which can potentially create bias; iv) being less perfect experiments than efficacy trials; v) sacrificing internal validity to achieve generalizability; and vi) often requiring large sample sizes to detect small treatment effects in heterogeneous populations. In this paper, we discuss whether these criticisms hold merit, or if they are simply driven by confusion about the purpose of pragmatic trials. We use the Cardiovascular Health Awareness Program (CHAP) trial - a community randomized pragmatic trial designed to assess whether offering a highly organized, community-based CHAP intervention compared to usual care can reduce cardiovascular disease-related outcomes - to address these specific criticisms and illustrate how to reduce this confusion.

**Trial registration:**

Current controlled trials ISRCTN50550004 (9 May 2007).

## Background

The Canadian Institutes of Health Research (CIHR) defines knowledge translation (KT) as a “*dynamic and iterative process that includes synthesis, dissemination, exchange and ethically sound application of knowledge to improve the health of Canadians, provide more effective health services and products and strengthen the health care system*” [1]. Thus, KT is a quality improvement process whose ultimate goal is to promote the use of evidence to support practice and policy. KT researchers have noted that whereas KT promotes evidence-based practice (EBP), the methods used to promote EBP are not always necessarily evidence-based [2]. As such, randomized controlled trials of KT interventions play a significant role in the KT process. These KT trials are designed based on the following considerations: i) the right PICOT (population; intervention; comparator; outcomes; time-frame) research question that is relevant to patients, healthcare providers and policy-makers; ii) the right methodology, such as a randomized controlled trials (RCT), to minimize bias; iii) large sample sizes - to maintain sufficient power to account for heterogeneity of populations studied and variability in the estimated treatment effects; and most importantly iv) a pragmatic strategy in design, conduct and analysis - to optimize the relevance of the results for real world practice and decision-making.

## What is a pragmatic strategy?

The Oxford English Dictionary defines “pragmatic” as an adjective meaning *“Dealing with matters in accordance with practical rather than theoretical considerations or general principles; aiming at what is achievable rather than ideal; matter-of-fact, practical, down-to-earth”* [3]*.* Inspired by Schwartz and Lellouch’s work [4], a pragmatic approach requires that when comparing treatments in the “real world,” one should take into account:

1. The realities of life - that patients may change or withdraw treatment, may not adhere to treatment, and may experience barriers to treatment.
2. The patient, provider and policy-maker have preferences and pressures.
3. Practice variations among healthcare providers.
4. The existence of co-morbid conditions - patients may take multiple medications (which can interact with the treatment of primary interest).
5. Variations in decision-making processes between administrators or policy-makers, including funders.

A pragmatic trial is designed to answer the practical question of whether offering an intervention compared with some alternative (for example, usual care) within the real world environment does more good than harm. That is, a pragmatic trial addresses a question of whether an intervention does work under usual healthcare conditions in patients to whom it is offered [5, 6]. Other synonymous terms used for a pragmatic trial include *practical*, *management* or *effectiveness* trial. On the other hand, explanatory trials, also known as efficacy trials are designed to test whether an intervention can work under ideal, tightly controlled conditions in patients who receive it [5, 6]. They are therefore intentionally designed not to be pragmatic.

A key advantage of a pragmatic trial is that, if its result is positive (that is, the intervention is shown to cause more good than harm), one can be reasonably sure that the therapy being tested really works and can be successfully implemented in real practice. A disadvantage is that if its result is negative (that is, it is unclear whether the intervention does more harm than good), one cannot distinguish a worthless therapy from an efficacious therapy that is not applied or accepted widely enough by the participants. Hence, in pragmatic trials, it is better to test therapies that have already been shown to work in explanatory trials. On the other hand, the main advantage of explanatory trials is that if they are negative, one can abandon the therapy; a disadvantage is that if they are positive, one still does not know whether the therapy will work in usual healthcare conditions. Thus, positive explanatory trials still require therapies to be tested further in pragmatic trials, in order to assess their effects in real-world settings.

For some key resources on pragmatic and explanatory trials and the differences between them, we refer the reader to the clinician-trialist rounds by Dr. David Sackett [5, 7], where he reviews the main features of pragmatic trials, and MacPherson [6]. See Table 1 for additional resources on pragmatic trials and related issues.

**Table 1:** Administrative database sources used for the Cardiovascular Health Awareness Program (CHAP) evaluation

Database	Description of contents
The Census data	Contains the sociodemographic profile and a measure of deprivation associated with different regions (small regions called dissemination areas).
The Client Agency Enrolment Program	Tracks patient enrollment to individual family physicians.
The Corporate Provider Database	Captures physician demographic and training information, and tracks their enrolment in practices and the primary care service organizations.
The Discharge Abstract Database	Provides information on all acute care hospitalizations.
The National Ambulatory Care Reporting System (*NACRS*)	Provides information on all emergency department encounters and same day surgeries.
The Ontario Drug Benefit Program Database	Tracks all provincially reimbursed prescription drug products dispensed to Ontarians aged 65 years and older or receiving social assistance.
The Ontario Health Insurance Plan Claims History Database	Captures information regarding in-and-out patient physician services and outpatient diagnostic and laboratory services.
The Ontario Physician Human Resources Database	Identifies area of specializations for physicians and allows primary care providers who are generalists to be identified.
The Registered Person’s Database	Captures demographic information and vital status (alive/deceased) for all Ontarians insured under the *Ontario Health Insurance Plan*
The Statistics Canada’s Postal Code Conversion File	Allows patients to be attributed to a census dissemination area based on postal code.

In this paper, we will discuss some of the key criticisms and confusion around pragmatic trials, whether or not these criticisms hold merit, and use the Cardiovascular Health Awareness Program (CHAP) trial, a community randomization pragmatic trial designed to assess whether offering a highly organized, community-based CHAP intervention compared to usual care can reduce cardiovascular disease (CVD) related outcomes [8], to illustrate how to address the issues.

## Main text

### Confusions and controversies about pragmatic trials and how to deal with them

Pragmatic trials have been heavily criticized in the literature [9] for the following reasons: i) high rates of loss to follow-up; ii) nonadherence to study intervention; iii) unblinded treatment and patient self-assessment, which can potentially create bias; iv) being less perfect experiments than efficacy trials; v) sacrificing internal validity to achieve generalizability; and vi) often requiring large sample sizes to detect small treatment effects in heterogeneous populations. The first three (i-iii) suggest that pragmatic trials may lead to biased estimates of effect of therapies or interventions, whereas the last three (iv, v and vi) suggest that pragmatic trials may not be a good use of research funds or may be infeasible to conduct.

The key issue is whether these criticisms are a fundamental problem in real-world health care. Do real-world patients know what treatment they are supposed to take, but often do not take it as prescribed, drop out of care, etcetera? If so, the problem must be permitted to occur, in order to get a clear answer as to whether the underlying therapy works in the real world. The more significant challenge in pragmatic trials is to prevent the problem from leading to a biased conclusion.

In the next section, we describe our experiences in designing and conducting a KT trial using a pragmatic strategy - the CHAP trial designed to implement evidence-based guidelines to improve cardiovascular health among the elderly in Ontario communities - to illustrate how one can address these criticisms in the design, conduct and analysis of a pragmatic trial.

#### Ethics

The CHAP trial was approved by the research ethics boards at Bruyère Continuing Care in Ottawa, Sunnybrook Health Sciences Centre in Toronto, and McMaster University in Hamilton. Informed consent was obtained from each participant.

### Addressing the criticisms: the CHAP trial

Cardiovascular diseases account for about 30 % of all deaths around the world, with more than 50 % of deaths due to stroke and about 15 % attributable to hypertension [10]. Hypertension is one of the leading risk factors for death in North America [10]. It is a major risk factor for cardiovascular disease (CVD), and a leading cause of hospitalization, disability and death in North America [11, 12]. In Canada, the burden of CVD is huge - with one CVD or stroke death every 7 min. Cardiovascular disease accounts for 17 % of the total hospitalizations and over CAN $22 billion is spent each year on CVD problems [13]. We know from efficacy or explanatory CVD trials that a decrease of 10 mmHg in systolic blood pressure or 5 mm. Hg in diastolic blood pressure (with one medication or a change in lifestyle) significantly improve health outcomes: they can reduce heart failures by 50 %, strokes by 40 % and death or heart attacks by 10-15 % [14, 15]. However, we also know that major gaps exist in making efficacious therapies or interventions effective in the real world. For example, detection, treatment and control of hypertension remain sub-optimal, with only half of the people with hypertension actually being diagnosed; of those diagnosed, only half are treated, and of those treated, only half control their hypertension - a phenomenon that is seen in many diseases and referred to as the rule of halves [16]. Many people are unaware they have high blood pressure (BP), which is why hypertension is often called “the silent killer” [17]. Furthermore, recommended techniques for BP measurement are rarely followed, and community-based interventions are often not linked to primary care.

The CHAP study was a cluster randomization trial, with the community as the unit of randomization, intervention, analysis and inference, designed to address the CVD problems created by hypertension [8]. Its PICOT (population, intervention, control, outcomes and time-frame) question was as indicated below:

**Table:** 

• **P:** Among midsized Ontario communities,
• **I:** does a highly organized, community-based program that combines offering blood pressure assessments and recording of CVD risks to everybody > 65 years old with peer health volunteer-delivered education and referral of all new or uncontrolled hypertensives to a source of continuing care (CHAP session),
• **C:** compared with usual care (that is, absence of this community-based program),
• **O:** reduce community rates of hospitalization for acute myocardial infarction, stroke and congestive heart failure
• **T:** over 12 months?

Thirty-nine communities were selected, stratified by size and geographical location, and randomly allocated to intervention (20 communities) and control (19 communities) arms. A detailed description of the design and development of the CHAP trial has been published elsewhere, [18–21], along with the main results [8].

### How pragmatic was the CHAP trial?

**Fig. 1:**
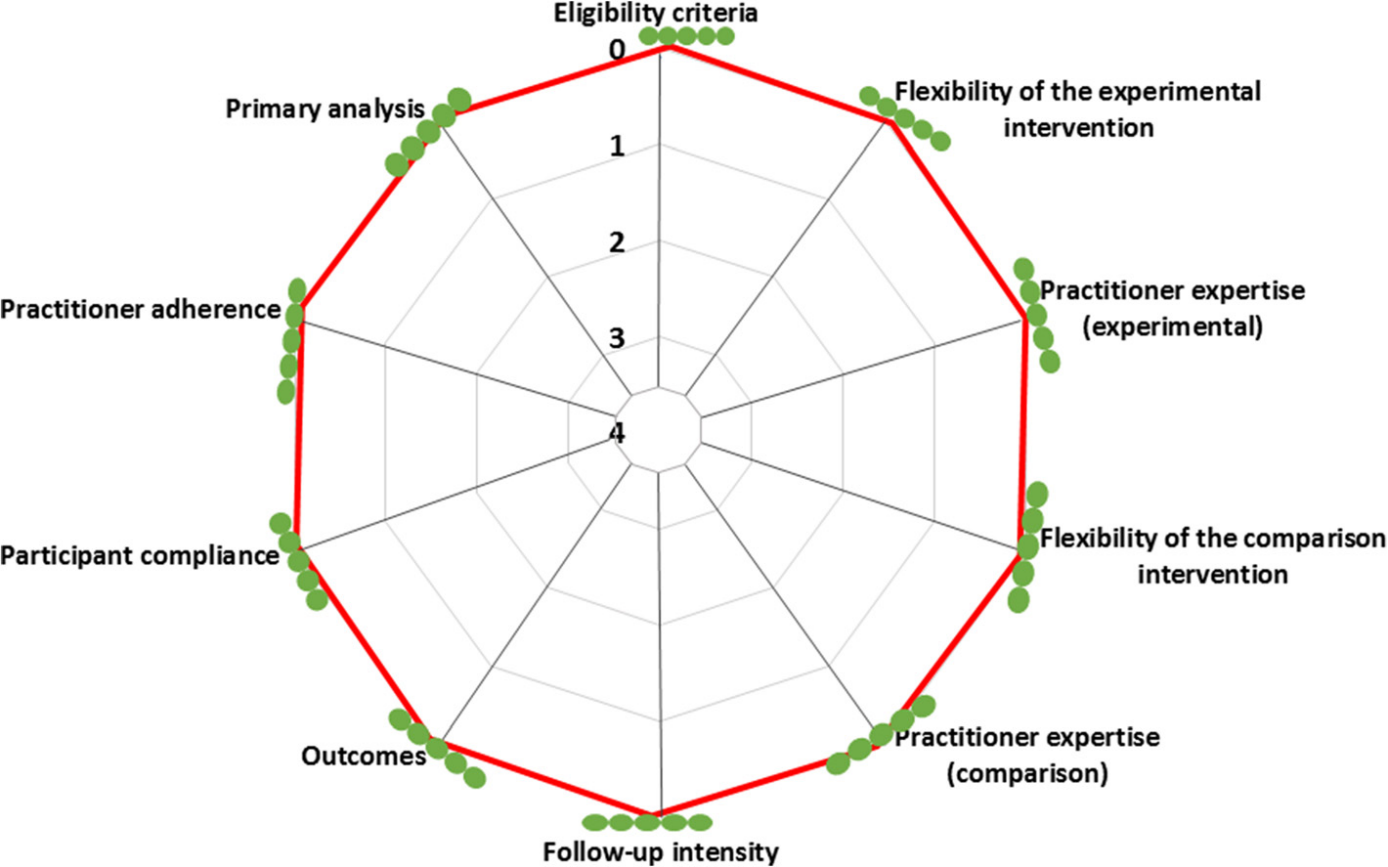
PRECIS score for the CHAP trial at the end of trial. The green dots represent the investigators’ scores along the pragmatic-explanatory continuum (4 = explanatory; 0 = pragmatic). All investigators scored the CHAP trial as pragmatic

Figure 1 shows the PRECIS (pragmatic-explanatory continuum indicator summary) diagram for the CHAP trial based on scoring by five CHAP investigators (Lisa Dolovich, Larry W Chambers, Lehana Thabane, Janusz Kaczorowski and J Michael Paterson). Introduced in 2009, the PRECIS is a tool that can be used to indicate the level of pragmatism of a trial on 10 elements [22]. The wider the web on the PRECIS wheel, the more pragmatic a trial design is, and the closer it is to the center of the wheel, the more explanatory it is. Judging from the PRECIS wheel for the CHAP trial, the CHAP investigators believed that CHAP was a highly pragmatic trial. Scoring was done independently by five investigators and the scores of each investigator are shown as a green dot in the Fig. 1.

The CHAP intervention was followed by a statistically significant 9 % relative reduction (RR) in our composite endpoint of hospitalization for acute myocardial infarction (MI), congestive failure or stroke. There were statistically significant reductions favoring the intervention communities in hospital admissions for acute MI and congestive HF, but not for stroke. Extrapolating to the general population aged 65 years, the 9 % RR means that the CHAP intervention would result in approximately 5,000 fewer hospital admissions for CVD annually in Ontario.

### How did we address the key criticisms about pragmatic trials in the CHAP trial?

#### Criticism 1: High rate of loss to follow-up

It is important to note that loss to follow-up is a fundamental feature of pragmatic trials because loss to follow-up is a normal process of clinical care in the real world. We prevented it from causing bias in CHAP by not adding artificial follow-up clinic visits for patients who did not attend. Instead, we used independent administrative databases that recorded the outcomes for all community dwelling residents, regardless of whether they participated in the program or not or whether they moved in, out or between communities during the study period (Table 1). We then applied the intention-to-treat (TT) principle to analyze the data, by including all residents aged 65 years or older in the control and intervention communities. In fact, ITT analysis is the most appropriate way to answer the question of whether an intervention works under usual conditions [6, 22].

#### Criticism 2: Non-adherence to study intervention

Non-adherence, a normal process in clinical care in the real world, is fundamental to pragmatic trials because they are designed to study real-world practice. Thus, “…*changes in individual patients’ treatment reflect normal clinical practice…*” [9]. We prevented it from causing *bias* in CHAP by using ITT analysis to describe the real-world situation. We did not adjust for whether or not the participant attended any of the CHAP sessions, followed up on the medical advice from their physicians, or adhered to their hypertension or CVD medications.

#### Criticism 3: Unblinded patients and physicians and patient self-assessments

Our intent was to preserve the ecology of normal care, where doctors know what they are prescribing and patients know what they are receiving (that is, people act as they would normally without the influence of knowing they are part of a trial). No one is blinded. Therefore, unblinding is fundamental to pragmatic trials *to “…preserve the ecology of care…”* [9]*.* Our strategy to prevent it from causing *bias* in CHAP involved independent adjudication of outcomes; measuring hospitalizations for CVD-related events that may be major consequences of hypertension. These were determined through administrative databases, not self-reports. Housed at the Institute for Clinical Evaluative Sciences (www.ices.on.ca), these databases include routinely collected administrative health data (see Table 2). The datasets have been assessed for reliability, completeness and validity, and have been widely used in health services research [23–26].

**Table 2:** Some key resources on pragmatic trials

Topic	Resource
The what, how, and why	1 Sackett DL. Clinician-trialist rounds: 16. Mind your explanatory and pragmatic attitudes! - part 1: what? Clin Trials. 2013; 10(3):495–8.
	2 Macpherson H. Pragmatic clinical trials. Complement Ther Med. 2004 Jun-Sep; 12(2–3):136–40.
	3 Sackett DL. Clinician-trialist rounds: 17. Mind your explanatory and pragmatic attitudes! Part 2: How? Clin Trials. 2013 Aug; 10(4):633–6.
	4 Staa TP, Goldacre B, Gulliford M, Cassell J, Pirmohamed M, Taweel A, et al. Pragmatic randomised trials using routine electronic health records: putting them to the test. BMJ. 2012; 344:e55.
	5 McMahon AD. Study control, violators, inclusion criteria and defining explanatory and pragmatic trials. Stat Med. 2002 May 30; 21(10):1365–76.
	6 Tunis SR, Stryer DB, Clancy CM. Practical clinical trials: increasing the value of clinical research for decision making in clinical and health policy. JAMA. 2003 Sep 24; 290(12):1624–32.
	7 Yoong SL, Wolfenden L, Clinton-McHarg T, Waters E, Pettman TL, Steele E, Wiggers J: Exploring the pragmatic and explanatory study design on outcomes of systematic reviews of public health interventions: a case study on obesity prevention trials. J Public Health (Oxf) 2014, 36(1):170–176.
Ethical issues pragmatic trials using cluster randomization	8 Hutton JL. Are distinctive ethical principles required for cluster randomized controlled trials? Stat Med. 2001 Feb 15; 20(3):473–88.
	9 McRae AD, Weijer C, Binik A, Grimshaw JM, Boruch R, Brehaut JC, et al. When is informed consent required in cluster randomized trials in health research? Trials. 2011; 12:202.
	10 Chaudhry SH, Brehaut JC, Grimshaw JM, Weijer C, Boruch R, Donner A, et al. Challenges in the research ethics review of cluster randomized trials: international survey of investigators. Clin Trials. 2013 Apr; 10(2):257–68.
PRECIS (pragmatic-explanatory continuum indicator summary) design tool and applications	11 Riddle DL, Johnson RE, Jensen MP, Keefe FJ, Kroenke K, Bair MJ, et al. The Pragmatic-Explanatory Continuum Indicator Summary (PRECIS) instrument was useful for refining a randomized trial design: experiences from an investigative team. J Clin Epidemiol. 2010 Nov; 63(11):1271–5.
	12 Thorpe KE, Zwarenstein M, Oxman AD, Treweek S, Furberg CD, Altman DG, et al. A pragmatic-explanatory continuum indicator summary (PRECIS): a tool to help trial designers. J Clin Epidemiol. 2009 May; 62(5):464–75.
	13 Zwarenstein M, Treweek S. What kind of randomized trials do we need? J Clin Epidemiol. 2009 May; 62(5):461–3.
	14 Maclure M. Explaining pragmatic trials to pragmatic policymakers. J Clin Epidemiol. 2009 May; 62(5):476–8.
	15 Tosh G, Soares-Weiser K, Adams CE. Pragmatic vs explanatory trials: the pragmascope tool to help measure differences in protocols of mental health
	16 Randomized controlled trials. Dialogues in clinical neuroscience. 2011; 13(2):209–15.
	17 Koppenaal T, Linmans J, Knottnerus JA, Spigt M. Pragmatic vs. explanatory: an adaptation of the PRECIS tool helps to judge the applicability of systematic reviews for daily practice. J Clin Epidemiol. 2011 Oct; 64(10):1095–101.
	18 Selby P, Brosky G, Oh PI, Raymond V, Ranger S. How pragmatic or explanatory is the randomized, controlled trial? The application and enhancement of the PRECIS tool to the evaluation of a smoking cessation trial. BMC Med Res Methodol. 2012; 12:101.
Reporting of pragmatic trials	19 Zwarenstein M, Treweek S, Gagnier JJ, Altman DG, Tunis S, Haynes B, et al. Improving the reporting of pragmatic trials: an extension of the CONSORT statement. BMJ. 2008; 337:a2390.
	20 Campbell MK, Piaggio G, Elbourne DR, Altman DG. Consort 2010 statement: extension to cluster randomised trials. BMJ. 2012; 345:e5661.
	21 Hopewell S, Clarke M, Moher D, Wager E, Middleton P, Altman DG, et al. CONSORT for reporting randomised trials in journal and conference abstracts. Lancet. 2008 Jan 26; 371(9609):281–3.

#### Criticism 4: Less perfect experiments than efficacy trials

Pragmatic trials, like the CHAP trial are evidently not designed to answer questions about efficacy, but are the ideal strategy for answering questions about effectiveness. Efficacy and pragmatic trials should not be compared in this way as they seek to answer entirely different questions. In the CHAP trial, the efficacy of blood pressure measurements, CVD risk assessment, education and referral were never in question but rather the effectiveness of organizing these services in a community setting.

#### Criticism 5: Compromising internal validity to achieve generalizability

For pragmatic trials, “internal validity” would be compromised if the real-world conditions were altered - that is, if no crossovers were allowed, patients or clinicians were blinded, or artificial follow-up visits were created to minimize loss-to-follow up. In fact, the trial *would then no longer* be pragmatic. A problem with this criticism is that the critics inappropriately judge the internal validity of a pragmatic trial by applying criteria that are appropriate for explanatory trials; this is a major source of confusion about pragmatic trials. We ensured the internal validity of the CHAP trial by preserving the natural process of care in the real world and by using other techniques - such as random allocation of the intervention - to minimize the potential for a bias in its conclusions.

#### Criticism 6: Large sample size requirements for small treatment effects

Finding small treatment effects in heterogeneous populations is vital to improving health care [27]. At a population level, small differences can lead to substantial improvements in health and decreases in resource utilization and costs. The key challenge raised by this criticism is that of *feasibility* [28]. We made CHAP *feasible* by the following: including *everyone* aged 65 years or over who lived in the target communities; using existing community resources such as lead community organizations to coordinate the CHAP program; recruiting community-based family physicians to send out letters to all their eligible patients, inviting them to attend BP clinics set-up at the local pharmacies, which were manned by community peer health volunteers and mentors to assess CVD risk and measure BP; relying on community health nurses to ensure quality control; and using existing administrative databases for outcome assessment. The economic analysis of the CHAP trial showed that the real cost was on average CAN $20 per eligible resident aged at least 65 years old, and cost neutral (that is, there were no statistically significant differences in resource utilization or cost for hospitalizations, emergency department and physician visits, or prescription drugs between the CHAP arm and usual care) [29]. In fact, comparing the per-patient cost of the CHAP trial to that of most CVD explanatory trials (at least CAN $5500 per patient) revealed that that CHAP was extremely inexpensive [30].

## Conclusions

In addition to the arguments made above, other researchers have demonstrated that outcomes in patients who receive care in the context of an explanatory RCT (ideal circumstances) are neither better nor worse than in patients who receive care in real world (or pragmatic) circumstances [31, 32].

In May 2012, Dr. Sackett organized a special session on “Confusions and controversies around pragmatic trials” at the Society of Clinical Trials annual meeting in Miami, USA. In his usual thought-provoking manner, he challenged the audience to think seriously about the basis for these common criticisms of pragmatic trials: “*Are pragmatic trialists dancing with the devil or is it simply the confusion about the question being asked*?” He gave the following analogy^1^:

**Table:** 

*“If a 320 km/hr Ferrari Spider works well on an Formula-1 circuit, but crawls along the M-25 (major highway in the UK) during the morning and evening traffic jams:*
*• an explanatory trialist would lament that testing the Ferrari on the M25 “lacks internal validity”*
*• a pragmatic trialist would conclude that the “Ferrari is a waste of money for a daily commute!”*

Results from explanatory and pragmatic trials contribute to the body of knowledge needed to inform healthcare practice and policy. Knowledge translation efforts that employ pragmatic trial methods are imperative, especially for optimal management of chronic health conditions. Pragmatic trials aim to assess the effectiveness of therapies or interventions under routine care (real-world) conditions. The key is to guard against biased conclusions. However, it is also important to think about “bias” and “validity” in the context of “effect under routine care.” Tampering with routine care conditions may actually lead to biased results on whether therapies work in the real-world settings.

## Endnote

^1^Dave Sackett attributed this to Tjeerd-Pieter van Staa, head of research and honorary professor of epidemiology at the London School of Hygiene and Tropical Medicine, London UK.

## Abbreviations

BP: blood pressure
CHAP: Cardiovascular Health Awareness Program
CIHR: Canadian Institutes for Health Research
CVD: Cardiovascular disease
EBP: evidence-based practice
HF: heart failure
ITT: intention to treat
KT: knowledge translation
MI: myocardial infarction
PICOT: Participants, Intervention, Comparator, Outcome, Timeframe
PRECIS: Pragmatic Explanatory Continuum Indicator Summary
RCT: randomized controlled trial
RR: relative reduction
